# Food-liking patterns are associated with nutrition knowledge among children aged 7–9 years: a nationwide cross-sectional study

**DOI:** 10.3389/fnut.2026.1898168

**Published:** 2026-08-31

**Authors:** Marta Jeruszka-Bielak, Jadwiga Hamulka, Ewa Czarniecka-Skubina, Joanna Myszkowska-Ryciak, Małgorzata Drywień, Krystyna Gutkowska

**Affiliations:** 1 Department of Human Nutrition, Institute of Human Nutrition Sciences, Warsaw University of Life Sciences SGGW, Warsaw, Poland; 2 Department of Food Gastronomy and Food Hygiene, Institute of Human Nutrition Sciences, Warsaw University of Life Sciences SGGW, Warsaw, Poland; 3 Department of Dietetics, Institute of Human Nutrition Sciences, Warsaw University of Life Sciences SGGW, Warsaw, Poland; 4 Department of Food Market and Consumer Research, Institute of Human Nutrition Sciences, Warsaw University of Life Sciences SGGW, Warsaw, Poland

**Keywords:** 3-point Likert scale, children, food-liking patterns, nutrition knowledge, primary schools

## Abstract

**Objective:**

To examine the association between nutrition knowledge (NK) and food liking (FL) among children aged 7–9 years.

**Methods:**

This cross-sectional study was conducted within the Junior-Edu-Żywienie (JEŻ) Project in Poland and included 14,548 first- to third-grade students. Children completed questionnaires assessing basic nutrition knowledge and liking of selected foods. Principal component analysis (PCA) was used to derive food-liking patterns, and General Linear Model (GLM) was performed to examine their associations with NK.

**Results:**

All food-liking patterns significantly predicted NK, with the strongest positive associations found for “less commonly liked recommended foods” (B = 4.171) and “fruit, vegetables, water & juices” (B = 3.379). Conversely, greater liking of “sugar-sweetened beverages” (B = −1.268) and “energy-dense discretionary foods” (B = −0.832) was associated with significantly lower NK scores. School grade, place of residence, and interest in nutrition-related topics were also independently associated with NK.

**Conclusion:**

Food-liking patterns are significantly associated with NK in early school-aged children. Identifying specific knowledge gaps and food preferences provides a valuable foundation for prospective research. Future longitudinal and intervention studies are needed to evaluate whether integrated school- and family-based education, combined with experiential learning (e.g., nutritional, culinary workshops), can effectively enhance children’s NK, promote healthier food choices, and ultimately improve dietary behaviors and nutritional status.

## 1 Introduction

Dietary behaviors established during childhood play a significant role in determining health outcomes across the life course. Incorrect early dietary patterns have been linked to an elevated risk of obesity, cardiometabolic disorders, and other diet-related diseases in later life, underscoring the significance of childhood as a pivotal window for preventive public health interventions. While environmental factors, including family practices and school environment, strongly shape children’s eating behaviors, it is crucial to recognize the active development of their dietary attitudes and preferences. Among these, FL is considered one of the strongest determinants of children’s dietary behavior (1–4).

Food preferences develop early in life and are shaped by repeated exposure to foods, sensory experiences, and social influences. Children often show a strong preference for highly palatable foods characterized by higher levels of sugar, fat, and salt, whereas foods perceived as less palatable, such as some vegetables, are more likely to be rejected. It is important to note that children’s preferences for food are not uniform. According to previous research, distinct patterns of FL can be identified within child populations, reflecting different degrees of alignment with dietary guidelines. Examining such patterns may provide valuable insights into variability in children’s attitudes toward food and help explain differences in dietary behaviors (5–7).

A variety of factors may contribute to the development of FL in childhood. In addition to sensory exposure and family influences, children are increasingly exposed to broader food environments, including marketing messages and social norms surrounding specific foods (8). Concurrently, children progressively develop cognitive abilities that enable them to comprehend information regarding nutrition and well-being. In this context, NK is considered an important component of children’s food-related competencies and is often viewed as a key element of nutrition or food literacy.

In principle, the possession of greater NK may support healthier food-related attitudes by enabling children to recognize the nutritional value of foods and better understand dietary guidelines. However, empirical evidence suggests that the relationship between NK and dietary behavior is complex. While higher knowledge levels are often associated with more positive attitudes toward healthy eating, knowledge alone does not necessarily translate directly into healthier food choices. As stated in the relevant literature, dietary behaviors are also shaped by environmental, social, and sensory influences (9–11).

Studies assessing children’s NK indicate that it is often incomplete and uneven across different aspects of dietary recommendations. While children are generally able to distinguish between healthy and unhealthy foods, such as fruits, vegetables, and sugar-sweetened beverages, they may encounter difficulties when evaluating more complex or processed products. Concepts such as appropriate portion sizes or the interpretation of nutrition information may also present challenges for children (12). Qualitative studies further suggest that children frequently categorize foods based on everyday experiences and socially transmitted messages rather than formal nutritional concepts (13).

Despite the growing interest in children’s NK and dietary behaviors, relatively few studies have explored how NK relates to food liking, particularly when considering patterns of liking across various food groups. These analyses can offer valuable insights, as food preferences often predict dietary habits and may serve as an important link between knowledge and food choices.

The age range of 7–9 years offers a particularly relevant period for examining these relationships. At this stage of development, children already possess emerging cognitive abilities that allow them to understand basic nutrition-related concepts and to report their attitudes toward foods. At the same time, their dietary attitudes and preferences are still actively forming. Therefore, understanding how NK relates to FL at this stage may provide useful insights for designing early nutrition education strategies.

Therefore, the present study aimed to examine the relationship between NK and FL among children aged 7–9 years. To capture the heterogeneity in children’s attitudes toward food, we identified and assessed patterns of declared FL in terms of their alignment with healthy eating guidelines. Additionally, the study explored whether selected sociodemographic characteristics were associated with children’s NK.

## 2 Materials and methods

### 2.1 Study design, participants, and recruitment

This study presents the results of large nationwide cross-sectional study conducted within the Junior-Edu-Żywienie (JEŻ) Project in the period from April 2022 to November 2023. All eligible primary schools across Poland’s 16 voivodeships were invited to participate via email. Of the 14,698 schools contacted, 2,218 agreed to participate (15.1% response rate). A two-stage cluster sampling design was used, with schools selected proportionally to voivodeships and place size, followed by random selection of classes. All students in selected classes were eligible to participate with parental consent. Of the children initially participating in the study (14,600), those who did not meet the inclusion criteria (age below 7 years, lack of parental consent) or had missing or incomplete data were excluded. The final sample for present study comprised 14,548 children aged 7–9 years from 140 primary schools across Poland (Supplementary Figure S1). The sample was comparable to the general Polish population in terms of sex and grade distribution but included a lower proportion of children from rural areas (14). Although the study covered all voivodeships, participation was voluntary; therefore, the sample should not be interpreted as statistically representative of the entire population of Polish children aged 7–9 years. More details on the Junior-Edu-Żywienie (JEŻ) Project are presented elsewhere (15).

The study protocol was approved by the Ethics Committee of the Institute of Human Nutrition Sciences, Warsaw University of Life Sciences, Poland (Resolution No. 18/2022). The guidelines of the Declaration of Helsinki were followed during the study. Written informed consent for a child’s participation was collected from parents before the study. Children were informed in age-appropriate language about the study procedure, and participation was voluntary.

### 2.2 Data collection

Children completed two paper-based questionnaires that referred to (1) basic nutrition knowledge (Nutrition Knowledge Questionnaire—NKQ) and (2) liking of selected foods (Food Preferences Questionnaire—FPQ). The NKQ and FPQ were developed for the purposes of the JEŻ Project and adapted to the cognitive abilities of children aged 7–9 years. The NKQ was intended as a brief criterion-referenced assessment of basic nutrition knowledge rather than as a unidimensional psychometric scale. The items intentionally covered several key areas relevant to Polish dietary guidelines for children, including food-group recommendations, diet-health relationships, eating behaviors, physical activity, and selected sustainability-related topics. The FPQ was designed to assess declared liking of selected foods commonly known to Polish children. Both questionnaires were reviewed by the research team, pilot-tested among early school-aged children, and modified to improve clarity, graphical support, and feasibility of classroom administration.

The NKQ consisted of 15 items that varied in the difficulty level. Three answers were available for all questions: one correct, one incorrect, and one “I do not know.” The FPQ included a list of 28 food products and a three-point smiley face Likert scale with the expression that reflects their experience with food—ranging from smiling, neutral, and sad faces meaning “I like,” “I am not sure,” and “I dislike” the food/food group, respectively. This scale was adopted from a five-point smiley face Likert scale and limited to three points because, in the preliminary studies, students had difficulty differentiating their emotions (16). FPQ included two additional questions: the first on interest in nutrition-related topics (one-choice question) and the second on the main sources of the child’s NK (multiple choice question).

Data were collected in the classrooms, in the presence of teachers and researchers, in an auditory manner. Firstly, a short instruction was given, then questions from NKQ were read aloud one by one, additional explanation was given if needed, and the children completed the questionnaire by themselves. After the completion of NKQ, the FPQ was distributed and explained shortly. Children marked their chosen answers individually. Teachers and researchers constantly supervised the whole process and helped the children when needed. Questionnaires were checked for completion and coded.

In the next stage, the state of NK was assessed. Correct answers marked in NKQ were scored 1 point, while others or lack of response were scored 0 points. Points were summed up for each respondent and expressed as a percentage of correct responses. The maximum number of available points equaled 15 points. The NK score was categorized to facilitate interpretation and to distinguish children with insufficient, sufficient, or good levels of nutrition knowledge using *a priori* approach (15, 17). The following ranges of points were used to define insufficient, sufficient, and good levels of NK, respectively: 0–5 points (<33.3% of correct responses), 6–10 points (33.3–66.7%), and 11–15 points (>66.7%).

### 2.3 Data analysis

The statistical analyses were performed using SAS 9.4 (Statistical Analysis Software, SAS Institute Inc., Cary, NC, USA). The chi-square test was used to analyze categorical data. Principal Component Analysis (PCA) was performed within an exploratory framework as a data-reduction procedure to identify empirical patterns of declared food liking. The components were derived based on Pearson correlation matrices with orthogonal Varimax rotation. Factor scores were generated using the regression method. The number of components to retain was determined using eigenvalues > 1.0, visual inspection of the scree plot, the Kaiser-Meyer-Olkin measure of sampling adequacy (overall MSA = 0.837), and the conceptual interpretability of the final solution. Items with low primary loadings or ambiguous cross-loadings with no clear contribution to an interpretable pattern were excluded from the model; therefore, the item ‘eggs’ was removed. The cumulative variance explained by the 7 retained factors was 46.2%. While a higher total variance is typical for strictly controlled environmental data, a cumulative variance within the 40–50% range is deemed robust and highly acceptable in psychological and dietary behavior literature due to the inherent multidimensionality of individual food preferences.

The component scores obtained for each participant were rescaled to facilitate interpretation using the min-max normalization method (subtracting the minimum value and dividing by the range), which bound the factor scores within a [0–1] interval. These normalized factors were subsequently utilized as continuous predictors of students’ NK in a General Linear Model (GLM). Additionally, sociodemographic covariates such as sex, grade, place of living, and interest in nutrition-related topics were incorporated into the model as adjusting variables to control for potential confounding. For all analyses, the significance level was set at 0.05.

## 3 Results

A total of 14,548 children participated in the study, including similar proportions of boys (51%) and girls (49%), as well as first- (32%), second- (34%), and third-grade (34%) students. Approximately half of the participants lived in rural areas or towns with fewer than 20,000 inhabitants, whereas one-quarter lived in cities with more than 500,000 inhabitants.

The mean percentage of correct responses in the NKQ was 61.7%. Overall, 34% of children were classified as having a good NK level, with significantly higher proportions among girls (36%), third-grade students (43%), and children living in the largest cities (37%; Table 1).

**Table 1:** Participant characteristics by NK level (*n* = 14,548).

Descriptive	n	NK Level			*p*-value*
		Insufficient (0–5 pts; *n* = 1,306)	Sufficient (6–10 pts; *n* = 8,241)	Good (11–15 pts; *n* = 5,001)	
Sex					
Girls	7,192	8.4	55.8	35.8	<0.001
Boys	7,356	9.5	57.5	33.0	
Grade					
First	4,711	12.2	62.5	25.3	<0.001
Second	4,895	9.1	56.4	34.5	
Third	4,942	5.8	51.3	42.9	
Place of living					
Village	2,832	8.2	58.1	33.7	0.025
City < 20 k	2,357	9.8	58.0	32.2	
City 20–100 k	3,382	9.4	56.9	33.7	
City 100–500 k	3,649	8.7	55.9	35.4	
City > 500 k	2,328	8.9	54.4	36.7	

NK, nutrition knowledge. Values are presented as %; *Chi-Square test of independence.

Children’s responses to individual NKQ items according to NK level are presented in Table 2. Overall, the highest percentages of correct responses concerned the need for daily physical activity (89%), the relationship between diet and health (80%), and the role of breakfast in providing energy for learning and playing (79%). Most children were also aware of the dietary recommendations for sweets (86%) and vegetable and fruit consumption (76 and 70%, depending on the item). In contrast, the lowest percentages of correct responses were observed for recommendations regarding grain products (34%), dairy products (49%), and fish (52%), as well as sustainability-related topics, including replacing meat with legumes (29%) and the recommended balance between animal- and plant-based foods (47%).

**Table 2:** Correct responses to individual questions by NK level in children aged 7–9 years (*n* = 14,548).

Question “Do you think …	Total (*n* = 14,548)	NK Level		
		Insufficient (0–5 pts; *n* = 1,306)	Sufficient (6–10 pts; *n* = 8,241)	Good (11–15 pts; *n* = 5,001)
… you should be physically active every day, e.g., ride a bike, dance, go for walks?”	89.2	56.6	89.0	98.1
… sweets can be eaten every day?”	85.7	57.7	84.0	95.8
… health and strength depend on what you eat?”	80.1	45.5	77.3	93.9
… eating breakfast before school will give you the energy to learn and play?”	78.7	41.6	75.9	93.1
… vegetables and fruit should be eaten at least 5 times a day?”	76.0	35.0	72.8	91.9
“What portion of a *Healthy Plate* should vegetables and fruits occupy?”	70.0	24.7	64.4	91.0
… nuts, pumpkin and sunflower seeds are good for health?”	64.4	22.3	57.4	86.9
“What grain product do you think is better for your health?”	60.5	17.8	51.6	86.3
… if you eat vegetables with dinner, you can skip them at other meals?”	59.3	22.7	50.5	83.4
… you should eat fish at least once a week?”	52.2	14.6	44.0	75.4
… eating fish improves memory and immunity?	50.5	12.9	40.0	77.7
… eating dairy products once a week is enough?”	48.9	20.0	40.7	70.1
… you should eat more animal products than plant products?”	46.9	15.8	37.0	71.3
“What percentage of a *Healthy Plate* should grain products (bread, cereal, rice) occupy?”	34.2	11.5	26.6	52.6
… meat and cold cuts can be replaced with beans, lentils or peas?”	28.7	9.5	22.2	44.5

NK, nutrition knowledge. Values are presented as %; *Chi-Square test of independence.

Overall, 69% of children reported an interest in nutrition-related topics, with a higher proportion among those with good NK (73%) than among those with insufficient (57%) and sufficient (67%) NK (Table 3). Parents or grandparents (77%) and school (75%) were the most frequently reported sources of nutrition knowledge, whereas each of the remaining five sources was identified by fewer than half of the participants.

**Table 3:** Interest in nutrition-related topics and sources of NK in children aged 7–9 years in total and by NK level (*n* = 14,548).

Descriptive	Total (*n* = 14,548)	NK level			*p*-value*
		Insufficient (0–5 pts; *n* = 1,306)	Sufficient (6–10 pts; *n* = 8,241)	Good (11–15 pts; *n* = 5,001)	
Interest in nutrition-related topics (yes)	68.5	57.4	67.4	73.3	<0.001
Sources of NK					
Parents/grandparents	76.5	76.4	75.7	77.7	0.031
School	74.9	73.8	74.3	76.3	0.021
Books	40.3	39.3	39.3	42.4	0.001
Television	36.6	38.6	35.5	38.0	0.005
Internet	33.3	30.9	33.5	33.6	0.164
Friends	20.9	19.6	21.1	21.0	0.469
Magazines	11.5	11.0	11.4	11.9	0.511

NK, nutrition knowledge. Values are presented as %; *Chi-Square test of independence.

EFA identified seven interpretable food-liking patterns covering 27 out of 28 foods that were named after the group components: “energy-dense discretionary foods”; “less commonly liked recommended foods”; “dairy & breakfast cereals”; “meat, cold cuts & fish”; “fruit, vegetables, water & juices”; “sugar-sweetened beverages”; and “starchy staple foods.” The names assigned to the factors should be understood as descriptive labels for empirically derived food-liking patterns. They do not imply that all foods within a factor have identical nutritional value. Some factors included foods with mixed nutritional profiles, and therefore the interpretation of these patterns should be based on their individual components. However, one pattern labeled “less commonly liked recommended foods” comprises health-promoting components such as legumes, wholegrain bread, cottage cheese, and groats. Conversely, two patterns, namely “energy-dense discretionary foods” and “sugar-sweetened beverages” consist exclusively of unhealthy foods and drinks.” Details on group components and factor loading values are shown in Table 4.

**Table 4:** Food-liking patterns according to factor analysis in children aged 7–9 years (*n* = 14,548).

Food-liking patterns (F)	Factors						
	1	2	3	4	5	6	7
F1 “Energy-dense discretionary foods”							
Sweets	0.749						
French fries/hamburgers/hot dogs	0.749						
Savory snacks	0.745						
Pizza	0.655						
F2 “Less commonly liked recommended foods”							
Legumes		0.623					
Wholegrain bread		0.601					
Cottage cheese		0.524					
Groats		0.516					
F3 “Dairy & breakfast cereals”							
Milk			0.639				
Sweet dairy desserts			0.591				
Breakfast cereals			0.537				
Natural yogurt			0.467				
Cheese			0.412				
F4 “Meat, cold cuts & fish”							
Meat				0.786			
Cold cuts				0.774			
Fish				0.492			
F5 “Fruit, vegetables, water & juices”							
Fruit					0.718		
Vegetables					0.609		
Fruit juices					0.517		
Water					0.406		
Vegetable juices					0.376		
F6 “Sugar-sweetened beverages”							
Energy drinks						0.747	
Carbonated sweet drinks						0.615	
Iced tea type drinks/flavored water						0.597	
F7 “Starchy staple foods”							
Pasta							0.607
White bread							0.592
Potatoes							0.499

Food-liking patterns according to NK are presented in Table 5. All FL patterns were significant predictors of NK in the regression model. Positive associations were observed for five food-liking patterns, with the strongest effect identified for “less commonly liked recommended foods” (B = 4.171; 95% CI: 3.892, 4.450), followed by “fruit, vegetables, water & juices” (B = 3.379; 95% CI: 2.969, 3.790). Smaller, but still significant positive associations were found for “meat, cold cuts & fish” (B = 1.379), “dairy & breakfast cereals” (B = 0.972), and “starchy staple foods” (B = 0.612). In contrast, unhealthy food-liking patterns were inversely associated with NK. Greater liking of “sugar-sweetened beverages” (B = −1.268; 95% CI: −1.554, −0.982) and “energy-dense discretionary foods” (B = −0.832; 95% CI: −1.105, −0.559) was associated with significantly lower NK scores. Among the covariates, grade, place of living, and interest in nutrition-related topics were significantly associated with NK. Compared with the first grade students (reference category), both second grade (B = 0.546, *p* < 0.001) and third grade students (B = 0.982, *p* < 0.001) had significantly higher NK scores. Compared with students living in large cities (>500 k inhabitants), those residing in cities with fewer than 20 k inhabitants (B = −0.302, *p* < 0.001) and in cities with 20–100 k inhabitants (B = −0.193, *p* = 0.004) had significantly lower NK scores. No significant differences were observed for students living in rural areas (*p* = 0.092) or in medium-sized cities (100–500 k inhabitants; *p* = 0.407). Self-reported interest in nutrition-related topics was positively associated with NK (B = 0.332, *p* < 0.001). In contrast, no significant difference in NK was observed between girls (reference category) and boys (B = 0.008, *p* = 0.859). The overall regression model was statistically significant (*F*(15, 14,532) = 141.13, *p* < 0.001) and explained 12.7% of the variance in NK.

**Table 5:** Association between NK level and food-liking patterns in children aged 7–9 years (*n* = 14,548).

Parameter	Unstandardized B (95% CI)	*p*-value
F2 “Less commonly liked recommended foods”	4.171 (3.892; 4.450)	<0.001
F5 “Fruit, vegetables, water & juices”	3.379 (2.969; 3.790)	<0.001
F4 “Meat, cold cuts & fish”	1.379 (1.113; 1.646)	<0.001
F3 “Dairy & breakfast cereals”	0.972 (0.647; 1.298)	<0.001
F7 “Starchy staple foods”	0.612 (0.249; 0.975)	0.001
F6 “Sugar-sweetened beverages”	−1.268 (−1.554; −0.982)	<0.001
F1 “Energy-dense discretionary foods”	−0.832 (−1.105; −0.559)	<0.001
Grade		
First	Ref.	Ref.
Second	0.546 (0.445; 0.647)	<0.001
Third	0.982 (0.879; 1.084)	<0.001
Place of living		
Village	−0.119 (−0.258; 0.020)	0.092
City < 20 k	−0.302 (−0.447; −0.158)	<0.001
City 20–100 k	−0.193 (−0.327; −0.060)	0.004
City 100–500 k	−0.055 (−0.186; 0.076)	0.407
City > 500 k	Ref.	Ref.
Interest in nutrition-related topics		
No	Ref.	Ref.
Yes	0.332 (0.241; 0.424)	<0.001
Sex		
Girls	Ref.	Ref.
Boys	0.008 (−0.077; 0.093)	0.859

Adjusted R^2^ = 0.127; F(15, 14,532) = 141.13; *p* < 0.001. NK, nutrition knowledge; F, Food-liking patterns; 95% CI, 95% confidence interval; model adjusted for grade, sex, place of living, interest in nutrition-related topics.

## 4 Discussion

This study examined whether food-liking patterns were associated with NK among early school-aged children. Patterns of FL were identified and evaluated according to their consistency with healthy eating recommendations. NK and its reported sources were also examined.

The present study demonstrated that a greater liking for the healthy food pattern designated as “less commonly liked recommended foods” was associated with higher NK among children aged 7–9 years. Notably, this pattern comprised legumes, wholegrain bread, cottage cheese, and groats, which were among the least preferred items by the study participants. Furthermore, positive associations with NK were identified for FL patterns that incorporated a mix of recommended and, to a lesser extent, non-recommended food groups. These included “fruit, vegetables, water & juices,” “meat, cold cuts & fish,” “dairy & breakfast cereals,” and “starchy staple foods.” Among these, the “fruit, vegetables, water & juices’ pattern can be explicitly classified as health-promoting. This alignment with Polish dietary guidelines supports the notion that a single serving of whole fruits or vegetables can be substituted with 100% juice in children’s diets. Conversely, greater liking for unhealthy food groups was associated with lower NK. Specifically, children reporting liking for the “sugar-sweetened beverages” and “energy-dense discretionary foods” patterns had significantly lower NK scores. These patterns include foods that dietary guidelines recommend limiting, such as sugar-sweetened beverages, sweets, fast food, and savory snacks. Taken together, these findings suggest that higher nutrition knowledge is associated not only with greater liking of recommended foods but also with lower liking for foods that should be consumed sparingly.

These relationships between FL and NK may be explained in several ways. On one hand, the “true” association may come from the “real” liking of foods that are generally perceived as less palatable, notably in pediatric populations (18, 19). Food liking should be interpreted as an affective component of children’s food-related attitudes rather than as a direct measure of dietary intake. In early school age, declared liking may reflect sensory familiarity, repeated exposure, home availability, parental modeling, peer influences and broader food environments (20, 21). Therefore, the association observed for the “less commonly liked recommended foods” pattern may reflect broader food socialization processes rather than a direct effect of nutrition knowledge on liking. Children who declared higher liking of legumes, wholegrain bread, cottage cheese and groats may have had greater exposure to these foods at home (or school), and these same environments may also support the development of basic nutrition knowledge. Repeated exposure to less palatable foods in a positive context may promote acceptance and increase the liking of those foods (21). Such family practices may be in turn related to higher parental NK and awareness (22–24). Children raised in families with higher nutrition literacy can acquire NK in an informal way, as nutrition issues may be discussed more often, e.g., while preparing and eating meals together (25, 26). Interestingly, family members were the most popular source of NK found in our study. Moreover, children with healthier attitudes (and liking of healthy foods) may seek out more nutrition information and further increase their knowledge (27). In addition, greater liking of unhealthy drinks and foods could be related to higher availability and accessibility of such products at home, parents’ behaviors (modeling) (20, 28, 29) which is observed more commonly in families with lower socioeconomic status (27, 30) and lower parental NK (31).

On the other hand, social desirability bias could operate there (32). Children aged 7–9 years may become “people pleasers” in a school-based survey context. Some of them could mark the responses “I like” for healthy or “I do not like” for unhealthy products because they assumed the teachers and researchers expected that. The consciousness of “healthiness” may even impact the taste evaluation in children. A study conducted by Soldavini et al. (5) showed that fourth- and fifth-grade children perceived cookies and crackers served in packages containing a nutrition claim as tasting better than those in packages without such claims, although products were identical.

Our study revealed that children’s knowledge was at a moderate level on average (61.7% of correct answers). Similar results were found by other researchers, e.g., for Canadian 5–8 grade students (63.5%) (33), and for Japanese 4–6 grade students (68.5%) (23), although lower than in Japanese 1–3 grade students (77.4%) (23); however, the results should be compared with caution due to different methods and questionnaires used. In our study NK varied across the themes and was the highest for recommendations related to physical activity, sweets, vegetables, and fruit. On the contrary, the dietary guidelines for other food groups like grain products, dairy, and fish were less known by children. The least acknowledged issues related to sustainability. Those differences might be explained to some extent by the food and nutrition content in the school curriculum. In Poland, in the second grade of primary schools, the core curriculum focuses on basic nutritional aspects, the Healthy Eating Plate (34), the sugar content in commonly consumed foods, and the health consequences of excessive sugar consumption. In the third grade, some contents are expanded and deepened, particularly healthy lifestyle guidelines are discussed, e.g., “eat regularly 5 meals a day”; “drink water frequently”; “be physically active at least one hour a day.” Moreover, various sources of protein and meat replacements are also analyzed. This explanation can also be supported by the results that children’s NK scores gradually increased from first to third grade. Our findings may indirectly confirm the effectiveness of school-based education in increasing NK among early school-aged children. However, we cannot rule out the influence of natural development in perception and understanding of questions, which also evolves with age.

In the present study, place of residence was independently associated with NK, although the observed pattern was not entirely linear. Children living in large cities (>500 k inhabitants) demonstrated significantly higher NK than those living in small and medium-sized towns (<100 k inhabitants), whereas no significant differences were observed for children residing in rural areas or in cities with 100–500 k inhabitants. These findings suggest that the relationship between the residential environment and NK is more complex than a simple urban – rural dichotomy. Previous studies have reported inconsistent findings regarding the association between place of residence and children’s NK. However, most of these studies compared only urban and rural populations, without considering differences across levels of urbanization. While some studies reported higher NK among children living in urban areas (35), others found higher NK among their rural counterparts (33, 36). The inconsistencies across studies may reflect differences in country-specific educational systems, socioeconomic conditions, and access to nutrition education and health promotion initiatives, rather than the degree of urbanization itself.

In the present study, no significant difference in overall NK was observed between girls and boys. Nevertheless, a higher proportion of girls was classified as having good NK than boys, suggesting a tendency toward better NK among girls that did not translate into a statistically significant difference in the overall distribution of NK. A recent systematic scoping review reported mixed evidence, with several studies demonstrating higher NK among girls, whereas others found no significant differences between girls and boys, particularly in younger age groups (37). Likewise, a systematic review by Thakur and Mathur (38) concluded that the association between sex and NK is inconsistent across populations and generally weaker than the influence of other individual and environmental determinants.

Overall, a high proportion of children reported an interest in nutrition-related topics (68.5%), particularly those with a good NK level (73.7%). A similar level of interest in nutrition and health has been reported among Swedish adolescents (27). In our study, parents or grandparents (77%) and school (75%) were the most frequently reported sources of nutrition knowledge. Interestingly, only one-third of participants identified the Internet as a source of such knowledge. These findings are consistent with previous evidence showing that parents and teachers are the primary sources of nutrition information for younger children. In contrast, the Internet, particularly social media, becomes a more important source in older age groups (27), when smartphones are more widely available than during the early years of primary school.

Several limitations to this study are noted. Firstly, the study was cross-sectional in nature and selection was voluntary and not random. Selection bias cannot be excluded, because schools and children participated voluntarily. Schools more interested in nutrition education may have been more likely to participate in the project. Secondly, children were recruited through schools and were therefore nested within school environments. The present analysis did not include multilevel modeling or school-level random effects, and detailed school- or class-level characteristics were not included. Consequently, school-level influences and clustering effects cannot be excluded. The results should therefore be interpreted primarily in terms of the direction, magnitude, and plausibility of associations rather than as evidence of school-independent effects. Thirdly, given the large sample size, even small differences may reach statistical significance. Therefore, the results should be interpreted with attention to effect size, direction of association, and public health plausibility. Additionally, we measured the declared FL with a three-point smile face Likert scale adapted to the cognitive abilities of young children, while the tasting method is perceived as the most effective in measuring children’s food preferences (39). Although such approach of using a three-point ordinal response scale improved feasibility and comprehension among young children, it may have limited variability in responses and may have influenced the component structure. Future studies should verify these patterns using confirmatory methods and approaches specifically designed for ordinal data. Even though the questionnaires were age-adapted, graphically supported, and pilot-tested, they were not formally validated against external criteria and test–retest reliability was not assessed. This may limit comparability with studies using other instruments. Therefore, the results should be interpreted as declared nutrition knowledge and declared food liking assessed using study-specific instruments. Moreover, social desirability bias may have influenced both NKQ and FPQ responses. Children completed the questionnaires in a school setting and knew that the questions concerned nutrition. Therefore, some children may have reported liking foods perceived as healthy or disliking foods perceived as unhealthy because they considered such answers expected by adults. This may partly explain the observed associations between NK and declared food liking. Although the supervised classroom procedure improved standardization, comprehension, and completeness of responses in young children, it may also have increased the likelihood of socially desirable answers. Finally, residual confounding should be considered an important limitation of the present study. Variables such as parental education, household socioeconomic status, parental nutrition knowledge, home food availability, school characteristics, region, body weight status, dietary intake, and diet quality were not included in the model, although they may influence both nutrition knowledge and declared food liking.

Nonetheless, our study adds some insight into the relationship between FL and NK in early school-aged children. The large sample size and a nationwide character of the survey compose the strength of the study. The method of data collection (in the classroom with the supervision of teachers and researchers) also increases reliability. The atmosphere created during the data collection was relaxed and far from ordinary school lessons, which raised students’ willingness to participate and commitment to tasks. Both questionnaires were age-appropriate and appealing to the participants. NKQ included 15 questions that focused on specific content areas of nutrition, especially Polish dietary guidelines, including sustainability issues (33). The percentage of correct answers varied substantially across items, from 28.7% for replacing meat and cold cuts with pulses to 89.2% for daily physical activity, indicating that the NKQ included items of different difficulty. In turn, the FPQ contained the food products typical for Polish cuisine. Illustrations for each food group helped children recall and imagine the foods.

## 5 Conclusion and public health implications

This study demonstrated that declared food-liking patterns were associated with nutrition knowledge among children aged 7–9 years in Poland. While the cross-sectional design precludes drawing causal relationships or definitively establishing educational efficacy, these findings offer valuable insights for designing prospective research. Specifically, future longitudinal and intervention studies should examine whether integrated school- and family-based nutritional interventions, combined with repeated exposure to recommended foods, can effectively enhance both NK and healthier food-related attitudes.

Our descriptive analysis further revealed critical knowledge gaps among children regarding core dietary guidelines—particularly for grain products, dairy, fish, and sustainability issues. These gaps indicate key target areas for future trial designs. For instance, subsequent intervention research could evaluate whether educational strategies targeting the broader family structure (including parents and grandparents) successfully alter the home food environment by increasing the availability of healthy options. Similarly, prospective studies are needed to evaluate the measurable impact of school-based education, specifically focusing on how structural frameworks and dietary guidelines affect children’s long-term health and academic outcomes. Furthermore, experimental research designs, such as culinary workshops and sensory tasting sessions, could test the efficacy of experiential learning in fostering acceptance of texturally complex or less traditionally favored items like legumes, wholegrain bread, groats, and vegetables. Ultimately, long-term intervention trials remain essential to fully map how food liking and NK interact to shape objective dietary behaviors and nutritional status in early school-aged children.

## Data Availability

The datasets presented in this article are not readily available due to ethical concerns. Requests to access the datasets should be directed to project manager, Krystyna Gutkowska (krystyna_gutkowska@sggw.edu.pl) and corresponding author Jadwiga Hamulka (jadwiga_hamulka@sggw.edu.pl).
