# Supplementary material for: Food-liking patterns are associated with nutrition knowledge among children aged 7–9 years: a nationwide cross-sectional study
Source: Front Nutr. 2026 Aug 31;13:1898168. doi: 10.3389/fnut.2026.1898168 (PMC13572107; doi:10.3389/fnut.2026.1898168)
Supplement: Supplementary file 1 [file Supplementary_file_1.docx]

Supplementary Material

Food-liking patterns are associated with nutrition knowledge among children aged 7-9 years:
a nationwide cross-sectional study.

Marta Jeruszka-Bielak, Jadwiga Hamulka*, Ewa Czarniecka-Skubina, Joanna Myszkowska-Ryciak, Małgorzata Drywień and Krystyna Gutkowska

**Supplementary Figure 1.** Flowchart: study design and data collection.
